# Cross-national data sample on the environmental affection and cognition of adolescent students of varying interests in ecosystem services and sustainability

**DOI:** 10.1016/j.dib.2018.12.019

**Published:** 2018-12-13

**Authors:** Ibrahim Niankara

**Affiliations:** College of Business, Al Ain University of Science and Technology, Abu Dhabi, UAE

## Abstract

This article contains cross-national data on the environmental affection and cognition of adolescent students of varying levels of interest in ecosystem services and sustainability, socio-economic and demographic status, and media consumption patterns. The underlying source is the 2015 publicly released student questionnaire data file of the “Programme for International Student assessment (PISA)” (OECD, 2016) , which was used in the studies “Interest in the biosphere and students’ environmental awareness and optimism: A global perspective” (Niankara and Zoungrana, 2018) and “Scientific media dieting and students’ awareness and expectations about the environmental issues of deforestation and species extinction in the middle east and north America: An integrated cross cultural ecologic-economic analysis” (Niankara, 2018).

By providing information on 7 major environmental issues (including deforestation, species extinction, air pollution, water shortage, greenhouse gas emission, genetically modified organism, and nuclear waste) in contemporary societies, for 18,7821 students from 50 countries worldwide, this data provides a unique opportunity to investigate various aspects of the world youth population׳s contribution to national sustainability and disease prevention initiatives.

**Specifications table**

**Table:** 

Subject area	*Economics*
More specific subject area	*Ecological, Environmental and Health Economics*
Type of data	*Table*
How data was acquired	*From the publicly released student questionnaire data file of the 2015 Program for International Student assessment (PISA)*[1]
Data format	*R formatted data set*
Experimental factors	*Measures of Adolescent Students’ Environmental Awareness, Environmental Expectation, Interest in the Biosphere, Interest in Science as a means for disease prevention, Social, Economic and Demographic factors*
Experimental features	*Cross-sectional cross-country probability sample with respondent level final weights for global PISA representativeness.*
Data source location	*Original data collection covered students from 35 OECD countries and 37 partner countries, but the final treated data covers a total of 50 countries (listed in*Table 1*).*
Data accessibility	*The data is with this article*
Related research articles	*Niankara, I. Zoungrana, D.T. ‘Interest in the Biosphere and Students Environmental Awareness and Optimism: A Global Perspective’ Global Ecology and Conservation (in Press)*[2]
	*Niankara, I. (2018),’Scientific Media Dieting and Students Awareness and Expectations about the Environmental Issues of Deforestation and Species Extinction in the Middle East and North America: An Integrated cross Cultural Ecologic-Economic Analysis’, Preprints, pp. 1–33, (doi: 10.20944/preprints201804.0267.v1)*[4].

**Value of the data**

• This data allows researchers to examine the determinants of adolescent students’ environmental affection and cognition in the form of their awareness and expectations about seven major environmental issues including, deforestation, plant and animal species extinction, air pollution, water shortage, greenhouse gas emission, genetically modified organism, and nuclear waste.
• By covering adolescent students’ from 50 countries and economies over the world, the data allows for not only single country investigations of any or all of the above environmental issues, but also much richer cross-country comparative analyses.
• In times of global commitments to sustainability, which requires nations to take into account the social and environmental aspects of their quest for economic growth and development, this data provides a unique opportunity to investigate the potential contribution of digital media to national sustainability and disease prevention initiatives among the world youth population.

## 1 Data

This data article relies on information derived from the 2015 Programme for International Student Assessment (PISA) [1]. PISA is the triennial survey of adolescent students around the world, lunched by the Organization for Economic Co-operation and Development (OECD), to assess the degree of preparedness of students near the end of their compulsory education, for full participation in modern societies. The 2015 PISA provides five main data files including a student-questionnaire data file, a school-questionnaire data file, a teacher-questionnaire data file, a cognitive item data file and a file with questionnaire timing data.

Table 1 shows the frequency and percent relative frequency distributions of students across the 50 countries contained in the data.

**Table 1:** Frequency and percent relative frequency distributions of students.

N 187821	Absolute frequency of students	Relative frequency of students in percent
Australia	6613	3.52%
Austria	2763	1.47%
Belgium	1817	0.97%
Brazil	5704	3.04%
Bulgaria	2497	1.33%
Canada	10,476	5.58%
Chile	3286	1.75%
Chinese Taipei	4562	2.43%
Costa Rica	3190	1.70%
Croatia	3162	1.68%
Czeck Republic	3708	1.97%
Denmark	2235	1.19%
Dominican Republic	1594	0.85%
Estonia	3560	1.90%
Finland	3223	1.72%
France	2962	1.58%
Germany	1459	0.78%
Greece	3306	1.76%
Hong Kong	2440	1.30%
Hungary	2438	1.30%
Iceland	1836	0.98%
Ireland	3169	1.69%
Italy	6388	3.40%
Korea	3978	2.12%
Latvia	2646	1.41%
Lithuania	3435	1.83%
Luxembourg	2338	1.24%
Macao	3023	1.61%
Mexico	4308	2.29%
Montenegro	2222	1.18%
Netherlands	2649	1.41%
New Zealand	1980	1.05%
Peru	3589	1.91%
Poland	2939	1.56%
Portugal	2964	1.58%
Qatar	4965	2.64%
Russian Federation	3198	1.70%
Singapore	3997	2.13%
Slovak Republic	3061	1.63%
Spain	3416	1.82%
Sweden	2698	1.44%
Switzerland	2180	1.16%
United Arab Emirates	6919	3.68%
Tunisia	1633	0.87%
Turkey	3478	1.85%
United Kingdom	6409	3.41%
United States	3197	1.70%
Uruguay	2095	1.12%
B-S-J-G (China)	5324	2.83%
Spain (Regions)	16,792	8.94%

Table 2 shows the descriptive statistics (mean and standard deviations) for select key socio-demographic quantitative measurements, while Table 3 presents the descriptive statistics (Absolute frequency and Relative frequency in percent) for select key socio-demographic qualitative measurements.

**Table 2:** Descriptive statistics for select key socio-demographic quantitative variables.

**Quantitative variables**	**Description**	**Mean**	**s.d.**
AGE	The age of the student.	15.79	0.29
MISCED	mother education level, based on international standard classification	4.24	1.65
FISCED	Father education level, based on international standard classification	4.2	1.66
hisei	Index of highest parental occupational status	53.01	21.82
ESCS	Standardized PISA Index of economic, social and cultural status.	−0.04	1.03
W_FSTUWT	Student final weight in the Data	47.51	99.56
CNTRYID	Unique Country Identifier for each of the 50 countries in the Sample		

Note: s.d. represents the standard deviation of the quantitative measurement

**Table 3:** Descriptive statistics for select key socio-demographic qualitative variables.

**Qualitative variables**	**Levels**	**Abs. Freq.**	**Rel. Freq.**
Gender	F- female	97,203	51.75%
	M-male	90,618	48.25%
IMMIG	Student׳s Immigration status		
	1-Native	162,265	86.39%
	2-Second-generation	11,956	6.37%
	3- First-generation	13,600	7.24%
GradeLev	Student grade level in School		
	7th grade	657	0.35%
	8th grade	6112	3.25%
	9th grade	61,196	32.58%
	10th grade	100,897	53.72%
	11th grade	18,241	9.71%
	12th and 13th grade	718	0.38%
REPEAT	whether or not the student has ever repeated a grade		
	0-no	166,822	88.82%
	1-yes	20,999	11.18%

Note: Abs. Freq. and Rel. Freq. represent respectively the absolute frequency and relative frequency distributions of the qualitative variables

## 2 Experimental design, materials, and methods

The data presented in this article is extracted from the raw SAS (TM) version of the “student questionnaire data file”, which is provided as a zipped folder under the name “PUF_SAS_COMBINED_CMB_STU_QQQ” in [1]. This zipped folder with a total size of 411 Mega Bites (MB) contains four files, two of which are raw SAS format data files named respectively “cy6_ms_cmb_stu_qqq.sas7bdat” and “cy6_ms_cmb_stu_qq2.sas7bdat”, along with their respective description files “CY6_MS_CMB_STU_QQQ.sas7bdat.format.sas” and “CY6_MS_CMB_STU_QQ2.sas7bdat.format.sas”.

The presented data is specifically extracted from the file “cy6_ms_cmb_stu_qqq.sas7bdat”, using the attached R codes. Due to the big size of the raw data file, and the memory constraint imposed by R, the presented R codes were used to import only the subset of the data containing our variables of interest. The selected subset was then saved into an R object named “dataN”, with initially 84 measurements (variables) on 519,334 students. This initial data object was subsequently treated to obtain the final data set “dataMena” with 187821 students. This last version of the data is the one used in [2], [4], and presented further in this data article. Further details on the sampling design of the 2015 PISA are found in the OECD report [3].

Below we now describe how the measures of environmental awareness and expectations, along with the measures of interest in the biosphere (ecosystem services and sustainability) and science as a means for disease prevention, and the measures of media consumption, were constructed from the raw data file.

All data treatment, variable reformulation, and descriptive statistics were implemented within the R statistical Software [5]; (see the attached computer codes, for more details).

### 2.1 Self-expressed environmental awareness measures

**Fig. 1:**
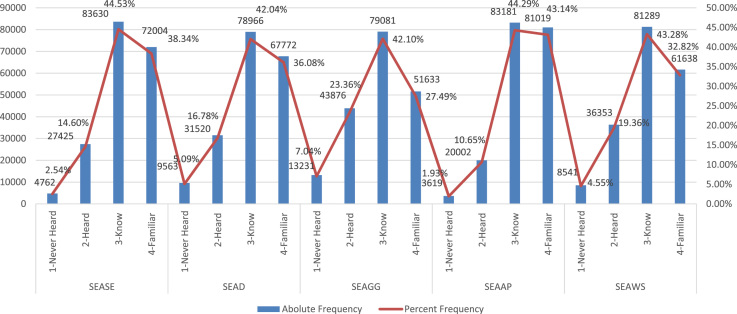
Absolute and Percent relative frequency distributions of adolescent students’ Environmental Awareness. Note: SEASE, SEAD, SEAGG, SEAAP, SEAWS: represent respectively students’ self-expressed awareness about the issues of Plant and Animal Species Extinction, Deforestation, Greenhouse gas emission, Air Pollution, and Water Shortage. They are all qualitative ordinal measurements, with four levels ranging from 1-never heard, 2-heard but can’t explain, and 3-know and can provide general explanation, and 4-Familiar and can provide detail explanation.

These are graphically summarized below in Fig. 1, with construction details provided in the attached R codes.

### 2.2 Self-expressed environmental expectations measures

**Fig. 2:**
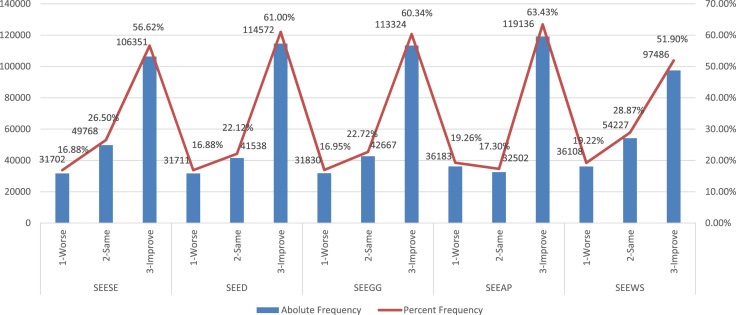
Absolute and Percent relative frequency distributions of adolescent students’ Environmental Expectation. Note: SEESE, SEED, SEEGG, SEEAP, SEEWS: represent respectively students’ self-expressed expectations about the issues of Plant and Animal Species Extinction, Deforestation, Greenhouse gas emission, Air Pollution, and Water Shortage. They are all qualitative ordinal measurements, with three levels ranging from 1-Worse, 2-Same, and 3-Improve.

These are graphically summarized below in Fig. 2, with construction details provided in the attached R codes.

### 2.3 Measures of interest in the biosphere and in science as a means for disease prevention

**Fig. 3:**
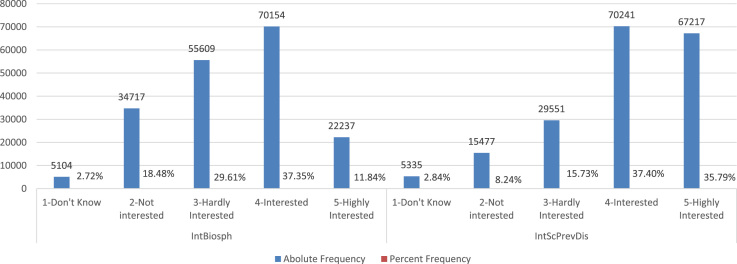
Absolute and Percent relative frequency distributions of students’ interest in the Biosphere and in Science for disease prevention. Note: IntBisph, IntScPrevDis represent respectively students’ self-expressed interests in the Biosphere (Ecosystem services and Sustainability) and in Science as a means for disease prevention. They are both qualitative ordinal measurements, with five levels ranging from 1-don’t know, 2-not interested, 3-Hardly interested, 4-Interested, and 5-Highly interested.

These are graphically summarized in Fig. 3 below with their construction details provided in the attached R codes.

### 2.4 Measures of weekly frequency of media consumption

**Fig. 4:**
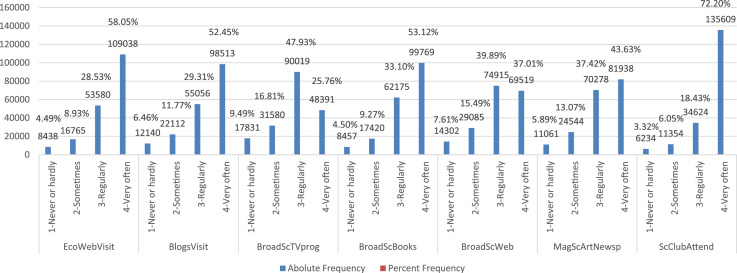
Absolute and Percent relative frequency distributions of students’ frequency of weekly Media Consumption. Note: EcoWebVisit, BlogsVisit, BroadScTVprog, BroadScBooks, BroadScWeb, MagScArtNewsp, ScClubAttend represent respectively students’ self-reported weekly frequency of Ecological website visits, news blogs visits, TV program watching on broad science, books reading on broad science, website browsing on broad science, magazines and science article reading, and science club attendance. They are all qualitative ordinal measurements, with four levels ranging from 1-never or hardly, 2-sometimes, 3-regularly, 4-very often.

See Fig. 4 below for a graphical summary of these variables, and consult the attached R codes for further details on their construction.
